# piRNAs from Pig Testis Provide Evidence for a Conserved Role of the Piwi Pathway in Post-Transcriptional Gene Regulation in Mammals

**DOI:** 10.1371/journal.pone.0124860

**Published:** 2015-05-07

**Authors:** Daniel Gebert, René F. Ketting, Hans Zischler, David Rosenkranz

**Affiliations:** 1 Institute of Anthropology, Johannes Gutenberg-University, Mainz, Germany; 2 Institute of Molecular Biology IMB, Mainz, Germany; CNRS UMR7622 & University Paris 6 Pierre-et-Marie-Curie, FRANCE

## Abstract

Piwi-interacting (pi-) RNAs guide germline-expressed Piwi proteins in order to suppress the activity of transposable elements (TEs). But notably, the majority of pachytene piRNAs in mammalian testes is not related to TEs. This raises the question of whether the Piwi/piRNA pathway exerts functions beyond TE silencing. Although gene-derived piRNAs were described many times, a possible gene-regulatory function was doubted due to the absence of antisense piRNAs. Here we sequenced and analyzed piRNAs expressed in the adult testis of the pig, as this taxon possesses the full set of mammalian Piwi paralogs while their spermatozoa are marked by an extreme fitness due to selective breeding. We provide an exhaustive characterization of porcine piRNAs and genomic piRNA clusters. Moreover, we reveal that both sense and antisense piRNAs derive from protein-coding genes, while exhibiting features that clearly show that they originate from the Piwi/piRNA-mediated post-transcriptional silencing pathway, commonly referred to as ping-pong cycle. We further show that the majority of identified piRNA clusters in the porcine genome spans exonic sequences of protein-coding genes or pseudogenes, which reveals a mechanism by which primary antisense piRNAs directed against mRNA can be generated. Our data provide evidence that spliced mRNAs, derived from such loci, are not only targeted by piRNAs but are also subject to ping-pong cycle processing. Finally, we demonstrate that homologous genes are targeted and processed by piRNAs in pig, mouse and human. Altogether, this strongly suggests a conserved role for the mammalian Piwi/piRNA pathway in post-transcriptional regulation of protein-coding genes, which did not receive much attention so far.

## Introduction

Small non-coding RNAs (sncRNAs or sRNAs) are involved in many cellular processes such as gene regulation, transposon repression and antiviral defense, which they realize by the principle of RNA interference [1]. To fulfill their functions all types of sRNA are dependent on Argonaute proteins, for which they act as guides that recognize targets based on sequence complementarity. Piwi-interacting RNAs (piRNAs, ~24–32 nt in length) represent a class of sRNAs that associate with Piwi clade Argonaute proteins, of which different species possess a varying number of paralogs [2–6].

The majority of piRNAs is organized in large genomic clusters, distributed throughout the genome at defined loci, ranging from 1–100 kb in size [3–6]. Further, piRNAs are characterized by a strong bias for uracil at the 5‘ end position (1U) and a preference for adenine at position ten (10A) for secondary piRNAs (see below). Finally, they are typically longer than miRNAs and siRNAs while displaying a broader size-distribution which is likely caused by the piRNA-specific 3’ end processing by exonucleases. These traits are the result of the biogenesis mechanisms of piRNAs which include two pathways [7–9]. In primary biogenesis, piRNAs are generated through the processing of long precursor transcripts into piRNA intermediates, which are loaded onto Piwi proteins that heavily select for 1U fragments [10], followed by 3’ trimming and 2‘-O-methylation of the 3‘ end by the methyltransferase Hen1 [11,12]. In secondary biogenesis, also known as ping-pong amplification loop, Piwi proteins loaded with primary piRNAs, target complementary transcripts, which are cleaved with a 10 nt offset from the 5’ end of the guiding primary piRNA to generate secondary piRNAs. Owing to this offset and the 1U bias of primary piRNAs, the resulting secondary piRNAs preferentially contain an adenine at the tenth position [7–9]. This 10 nt 5’ overlap of primary and secondary piRNAs is commonly referred to as ping-pong signature.

One of the major functions of the Piwi/piRNA pathway is the repression of transposable elements (TEs or transposons). Piwi proteins are primarily expressed in germ cells, regarding mammals especially during spermatogenesis [3,9]. In the course of spermatogenesis, genome wide demethylation, as part of the epigenetic reprogramming, leads to a reactivation of TEs [9,13]. In both mouse and fruit fly, mutations of Piwi proteins result in derepression of TEs in the germline leading to male sterility [14–17]. Similarly, deficiency of murine piRNA clusters results in an increased activity of TEs, emphasizing the importance of piRNAs in transposon silencing [18]. Accordingly, piRNA clusters are commonly perceived as transposon traps that acquire the capability of producing piRNAs directed against particular TEs as soon as the TE by chance jumps into such a locus [19].

Despite their important role in repressing transposon activity, in mouse only meiotically (pre-pachytene) expressed piRNAs are enriched for TE-related sequences, in contrast to pachytene piRNAs, of which only about 17% are TE-derived [3,4,14]. This led to the presumption that piRNAs might fulfill other functions besides TE silencing. Indeed, several studies in fruit fly suggested a role for piRNAs in regulation of protein-coding genes, including Stellate, vasa [20,21], Fasciclin 3 [22], and nanos [23]. Hints for a gene-regulatory function of the Piwi pathway in mammals have also been obtained in mouse [24,25], but neither the underlying mechanism, nor the discrete function has become clear so far.

The majority of mammalian species, including humans, possess a standard set of four paralogous Piwi proteins [26], while the bulk of research on mammalian Piwi/piRNA biology was conducted in mouse or rat, which express only three Piwi paralogs. In that sense, mice and rats might represent an exceptional realization of Piwi/piRNA biology. Hence, to investigate the nature of piRNAs in the mammalian germline in a context that resembles the regular condition with respect to Piwi protein equipment, we sequenced and analyzed testis expressed sRNAs of the pig, a species expressing all four mammalian Piwi paralogs. The pig is particularly interesting in the context of Piwi/piRNA biology, considering the unique TE landscape of the porcine genome, comprising e.g. active tRNA-derived short interspersed elements (SINEs) and pig-specific endogenous retroviruses (ERVs), while at the same time having a considerably lower share of TE sequences compared to other mammals [27]. Besides, porcine spermatozoa are known to exhibit extreme fitness due to domestic breeding and sexual selection in promiscuous mating systems resulting in sperm competition. Adding to the previous initial characterizations of porcine piRNAs [28,29], we focused on both, possible new aspects of the TE silencing function, as well as potential roles in the regulation of non-TE targets. Our present study strongly indicates that the mammalian Piwi/piRNA system is involved in post-transcriptional gene regulation and that piRNA clusters, which occupy a central role in this process, might be more dynamic and adaptable than previously thought.

## Methods

### Ethics statement

This study did not require approval by an ethics committee. Biological samples were obtained under current law from a licensed provider (Georg-August-University Göttingen, Animal Breeding and Genetics, Albrecht-Thaer-Weg 3, 37075 Göttingen, Germany).

### Preparation of sRNA libraries

Testis tissue was taken from an adult boar (*Sus scrofa domestica*) and stored at -80°C. Total RNA was extracted directly from testis tissue using TRI Reagent (Ambion) according to the manufacturer‘s instructions. The employment of 50 mg of tissue resulted in an RNA yield of approximately 140 μg. Total RNA was applied to a urea-based denaturing polyacrylamide gel (10%) together with the GeneRuler Ultra Low Range DNA Ladder and run for 20 minutes (1200 V, 50 mA, 60W). The 20–35 nt fraction was excised from the gel and resolved in 30 μl water using Amicon’s Ultrafree-MC and Ultra-0.5 3K centrifugal devices according to the manufacturer‘s instructions.

We portioned the obtained sRNA sample into two fractions and conducted sodium periodate treatment followed by ß-elimination with one of the two fractions according to the method applied by Rajasethupathy and colleagues with minor adjustments regarding the sample volumes [30]. A 5’-diphosphorylated and 3’-blocked RNA adapter (5’-rAppCTGTAGGCACCATCAATddC-3’, Integrated DNA Technologies) was directionally ligated to the 3’ end of periodate treated and untreated sRNA samples in absence of ATP using New England Biolabs T4 RNA Ligase 1 according to the following reaction mixture: 43 μl sRNA sample, 6 μl of 100% DMSO, 6 μl 10x NEB ligation buffer, 2 μl 3‘ RNA adapter, 2 μl T4 RNA ligase (10 U/μl) and 1 μl of RiboLock RNase Inhibitor (Thermo Scientific). The mixture was incubated at room temperature for 2 hours. For separation of sRNA molecules linked to a 3’ adapter we conducted acid phenol chloroform (Life Technologies) extraction and ethanol precipitation followed by separation of molecules ranging from 40 to 55 nt in length using polyacrylamide gel electrophoresis with subsequent gel extraction as described above.

A second RNA adapter carrying a 4 nt sequence tag and lacking a 5’-phosphate was ligated to the periodate treated (5’-GACUGGAGCACGAGGACACUGACAUGGACUGAAGGAGUAGAAA-3’) and untreated (5’-GACUGGAGCACGAGGACACUGACAUGGACUGAAGGAGAUCGAA-3’) sRNA samples in presence of ATP using New England Biolabs T4 RNA Ligase 1 according to the following reaction mixture: 36 μl sRNA sample, 3 μl RNA adapter, 6 μl 100% DMSO, 6 μl NEB 10x ligation buffer, 6 μl 10mM ATP, 2 μl T4 RNA ligase (10 U/μl), 1 μl RiboLock. The mixture was incubated at 37°C for 30 minutes.

The ligation reaction was stopped and RNA was purified by acid phenol chloroform extraction and ethanol precipitation and dissolved in water. Following cDNA synthesis using Superscript III Reverse Transcriptase (Life Technologies), the sample was PCR amplified (forward primer for periodate treated sample: 5’-ACATGGACTGAAGGAGTAGA-3’, forward primer for untreated sample: 5’-ACATGGACTGAAGGAGATCG-3’, reverse primer for both samples: 5’-ATTGATGGTGCCTACAG-3’) and ethanol precipitated. Both tagged samples were high throughput sequenced in parallel on an Illumina HiSeq 2000 system.

### Bioinformatic data processing and analysis

First, 5’ adapter and 3’ adapter sequences were clipped from NGS raw sequences and reads were allocated to periodate treated sRNA and untreated sRNA datasets based on the differentially tagged 5’ adapter. Considering a putative contamination by non-piRNA sequences, reads ranging from 18 to 34 nt in length were mapped in sense orientation to available ncRNA sequences from Ensembl database (release 77), miRBase [31] and the Genomic tRNA Database [32] using SeqMap [33] (version 1.0.12) to sort out sequences resembling microRNAs (miRNA) or fragments of other ncRNA types such as miRNA precursors, snRNA, snoRNA, rRNA and tRNA. Sequences that did not produce a match to any known ncRNA, thus representing putative piRNAs, were mapped to the genome of *Sus scrofa* (Sscrofa10.2.75) using SeqMap, taking only perfect matches into account.

To determine the amount of sRNA sequences related to TEs, the porcine genome was masked using RepeatMasker software and porcine transposon sequence data from Repbase [34]. The quantity of reads mapping to TEs was normalized for each sequence by the total number of genomic hits it produced. The identification and analysis of piRNA clusters was performed using the tracking and analysis software proTRAC [35] (version 2.0.2), searching for clusters with a minimum size of 10 kb, applying a sliding window size of 1 kb and an increment of 0.1 kb.

In order to identify cDNA sequences that exhibit a ping-pong signature, thus representing putative piRNA targets, we mapped sRNA sequences to annotated cDNA (Ensembl release 77). We applied a coverage threshold of 10 mapped sequence reads (counts were normalized by the number of hits per sequence) per one million mapped sequence reads to ensure comparability across the different probes that comprised different total numbers of sequence reads. The principals of this computational approach are described in Antoniewski 2014 [36].

To search for conserved cDNA targets of piRNAs in mammals, we applied identical procedures to human and mouse testis expressed sRNA datasets that are deposited at NCBI’s sequence read archive (SRA) under the accessions SRX271415, SRX271416 and SRX271417 for human sRNAs [37] and SRX154530 for mouse sRNAs [38]. We considered a ping-pong signature to be evident if the peak referring to the 10 nt overlap was at least 2-fold higher compared to the next highest peak. Generally, z-scores for ping-pong signatures were calculated according to the method applied by Zhang and coworkers [39]. The identified genes were subjected to GO term enrichment analysis [40], applying a *p*-value threshold of *p* = 0.05. To verify that the numbers of homologous genes targeted in different species is higher than expected by chance, we randomly sampled genes from two species according to the number of observed piRNA target genes (one million draws). We calculated expected values (E(X)) for the number of homologs that are present in both random sets based on the observed cross match. *P*-values correspond to the frequency of observed cases with a cross match equal or higher than observed for the original data set. The applied Perl scripts are available upon request.

### Data deposition

The complete sequence dataset is available at NCBI’s SRA under the following accessions: BioProject ID: PRJNA267635, Experiment: SRX761355, Run: SRR1654828.

## Results

### Annotation of porcine sRNAs

Overall 13,596,939 raw sequence reads were obtained from sequencing of periodate treated porcine testis RNA and subjected to several filtering and initial processing steps. Of 12,508,703 reads within the size range of 18–34 nt, 1,502,807 reads (12.0%) could be classified as miRNAs (0.09%) or fragments of other ncRNA species such as tRNA (11.6%), rRNA (0.09%), snoRNA (0.06%) and snRNA (0.01%), leaving 11,005,896 reads, comprising 3,226,011 non-identical sequences that represent putative piRNAs. A fraction of 7,219,711 reads, originating from 928,481 non-identical sequences, mapped perfectly to the genome of *Sus scrofa*, producing 24,579,193 genomic hits.

**Fig 1:**
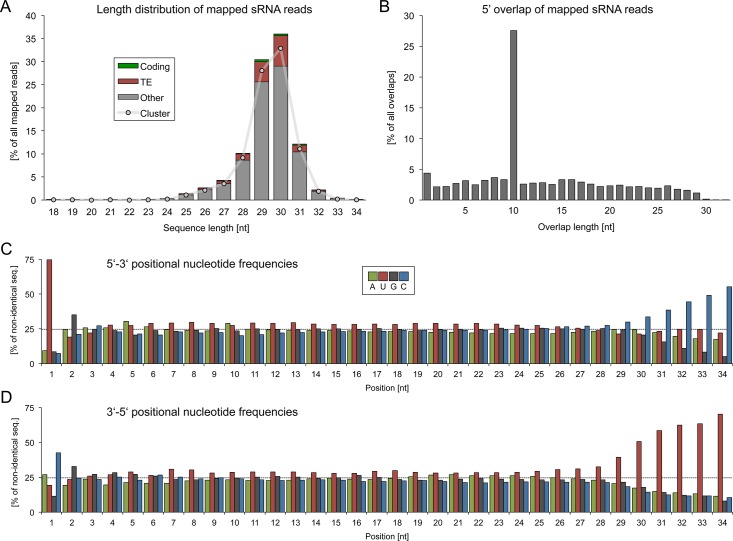
Basic characterization of putative piRNAs in porcine testes. (A) Length distribution of small RNAs. The mapped sRNA reads show an approximately Gaussian length distribution, ranging mostly from 24 to 33 nt with a peak at 30 nt. The majority of each size fraction maps to predicted piRNA clusters. (B) 5’ overlap of sRNAs. Sense and antisense sRNA reads produce a high rate of 10 nt 5’ overlaps. (C) Positional nucleotide frequencies starting from 5’ end. (D) Positional nucleotide frequencies starting from 3’ end.

The mapped sRNAs show a roughly Gaussian length distribution, ranging mainly from 24–33 nt with a peak at 30 nt (Fig 1A). More than 99% of all reads fall into the typical size range of mammalian piRNAs (24–32 nt) and the vast majority (91%) of sequence reads maps to one of 142 predicted piRNA clusters (see below). The mapped sRNA sequences exhibit a strong ping-pong signature, meaning a strong preference (z-score = 44.4) for 10 nt 5’ overlaps between sequences mapping to the sense and antisense strands of the genome, which is a hallmark of piRNAs, attributable to their specific biogenesis mechanism during the ping-pong cycle (Fig 1B, S1 File).

Another characteristic trait of piRNAs is constituted by a strong bias for uracil at the 5’ end and a consequential preference for adenine at the tenth position for secondary piRNAs. Nearly 75% of non-identical sequences start with a uracil, while adenine is only slightly enriched at position ten (29.2%), which suggests that the bulk of porcine pachytene piRNAs originates from primary processing (Fig 1C). Furthermore, we observed a bias for cytosine at the 3’ terminus (42.6%) and for guanine at the second position of both the 5’ (34.5%) and 3’ ends (32.9%) (Fig 1C and 1D).

Together, though we do not provide formal evidence for binding of these sRNAs to Piwi proteins, the overall characteristics of the analyzed sRNA dataset (size distribution, nucleotide composition, genomic clustering, ping-pong-signature) are in compliance with the typical piRNA traits and indicate a very low degree of contamination by non-piRNA sequences.

### TE-derived piRNAs

**Fig 2:**
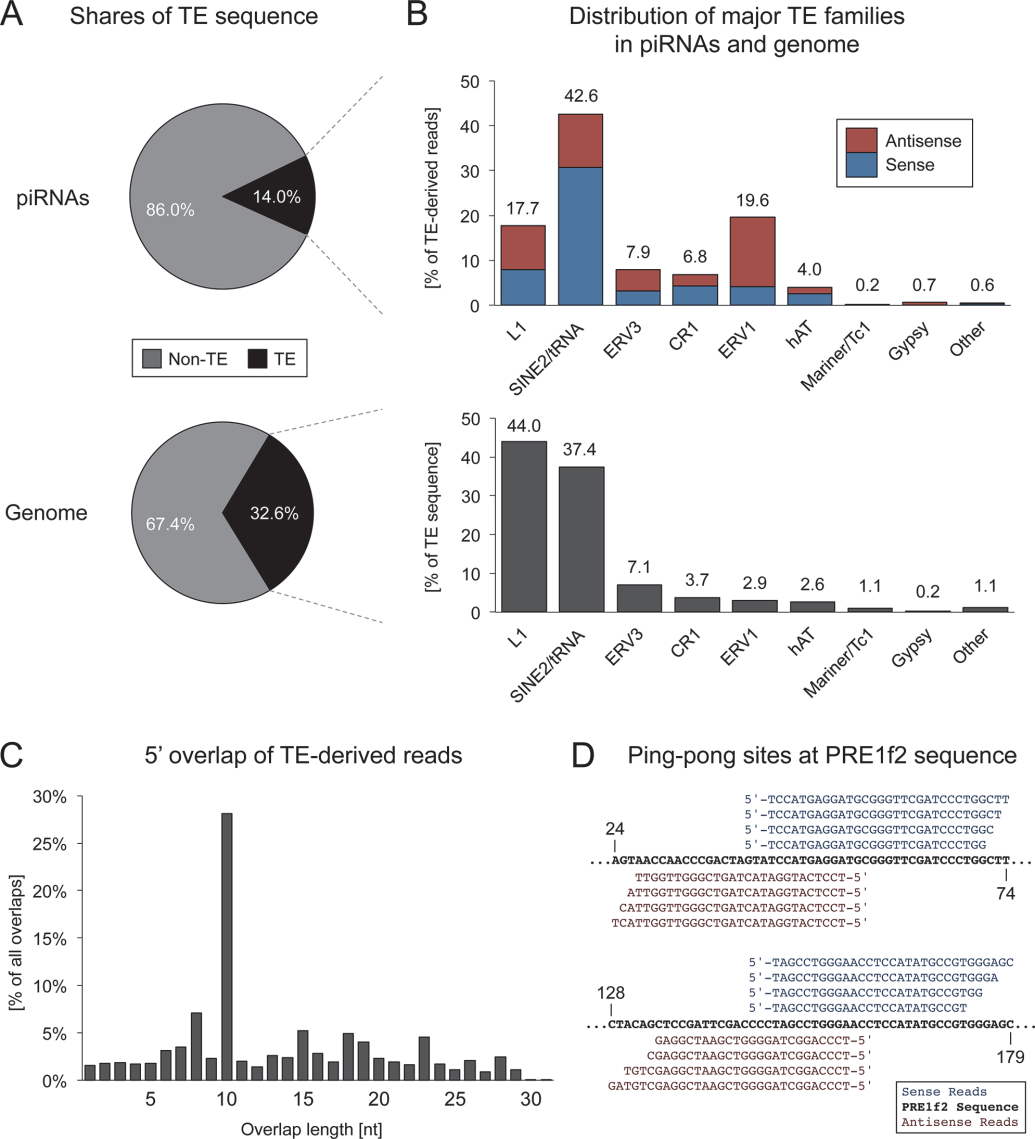
Transposon-derived piRNAs. (A) Shares of TE sequences in mapped piRNA reads and in the porcine genome. (B) Representation of TE families within the sequences of piRNA reads in sense and antisense direction compared to the genomic TE family distribution. (C) 5’ overlap of TE-derived piRNAs. (D) Mapping of piRNA reads to the sequence of a PRE1f2 element, a member of the tRNA-derived SINE subfamily of porcine repetitive elements. Regions from positions 24–79 nt and 128–179 nt are shown as exemplary target sites of ping-pong processing.

Transposon silencing is considered as the main function of piRNAs, hence the mapped piRNAs were screened for sequences that target genomic loci annotated as TEs. Overall 14.0% of total mapped reads (representing 16.3% of non-identical sequences) match transposon sequences (Fig 2A), of which SINEs contribute the largest proportion (5.9%), followed by LTR retrotransposons (4.0%), LINEs (3.5%) and DNA transposons (0.6%) (Fig 2B). Quantity and composition of TE-related piRNAs contrast the overall genomic situation with a total of 32.6% corresponding to TEs (Fig 2A), where the largest TE fraction is represented by LINEs (15.6%), followed by SINEs (12.2%), LTR transposons (3.5%) and DNA transposons (1.4%) (Fig 2B).

Though piRNAs generally map to TEs in both orientations and the overall amount of sense and antisense piRNAs is roughly equal, the sense/antisense ratio differs considerably for different transposon families (Fig 2B). While tRNA-derived SINEs like the abundant PRE elements show a strong bias for sense piRNAs, ERV1 elements exhibit a strong bias for antisense piRNAs. Since the majority of TE-related piRNAs originate from piRNA clusters, we assumed that these differences might result from insertional strand bias. Therefore we checked the insertion direction of TEs relative to the transcribed piRNA cluster strand. Indeed, we found that the insertion direction correlates well with, and thus can explain the different sense/antisense piRNA ratios for the most prominent TE classes, namely tRNA-derived SINEs, L1 and ERV1 (S1 Fig), which comprise more than three quarters of all TE-derived piRNA reads.

In order to search for evidence of ongoing TE repression via the ping-pong cycle, we analyzed the 5’ overlaps of sense and antisense piRNAs mapped to TE sequences (Fig 2C and 2D). Though we observed a marked ping-pong signature (z-score = 17.3) for TE-related piRNAs, indicating Piwi-dependent processing, both sense and antisense piRNAs show a strong 1U bias (84% and 87%, respectively), and only a slight elevation for 10A (38%) can be observed for antisense TE reads. This is in line with previous findings from mouse [9], where 1U-biased primary piRNAs generated from TE transcripts target piRNA cluster transcripts resulting in secondary 10A-biased antisense piRNA. Together, our data suggest that a noticeable fraction of antisense piRNAs originates from secondary processing while still most pachytene piRNAs are generated via the primary processing mechanism.

### Gene-derived piRNAs

**Fig 3:**
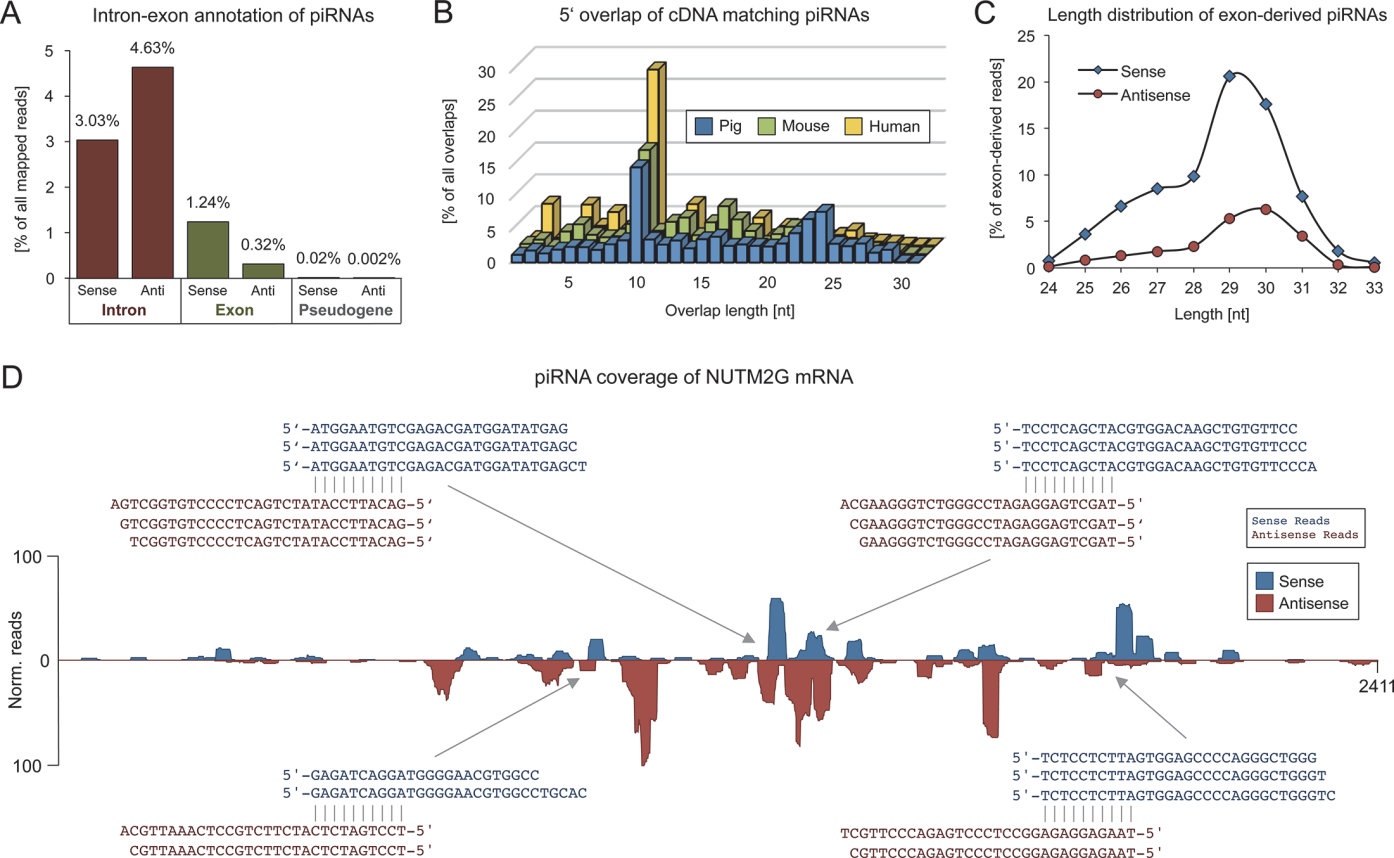
Gene-derived piRNAs. (A) Portions of piRNA reads mapping to introns, exons and pseudogenes in the porcine genome. (B) 5’ overlap of testis piRNAs from pig, mouse and human, mapping to corresponding annotated cDNA. In all three species a high rate of 10 nt 5’ overlaps is detectable. (C) Length distribution of sense and antisense exon-derived piRNAs. (D) Mapping of piRNA reads to the mRNA sequence of the protein-coding gene NUTM2G. Exemplary sites with 10 nt 5’ overlap between sense and antisense piRNA reads are indicated by arrows.

Gene-derived piRNAs were previously observed in diverse species but were generally considered to represent a byproduct derived from mRNAs that accidentally fall into the clutches of the Piwi/piRNA pathway, mainly because only sense piRNAs could be found. To investigate a potential impact of piRNA function on protein-coding genes, mapped piRNA reads were initially screened for sequences mapping to annotated coding DNA (cDNA). In total 1.8% of mapped reads, representing 9.4% of non-identical sequences, produce perfect matches to porcine cDNA. Intriguingly, when focusing on protein-coding genes we found that 7.6% of piRNA reads map to intronic sequences in sense (3.0%) and antisense (4.6%) orientation, which apparently cannot be explained by processing of spliced mRNA. Further, 1.6% map to exonic regions in sense (1.24%) and antisense (0.32%) orientation and 0.02% of piRNA reads match pseudogenes mainly in sense direction (Fig 3A).

To determine whether sRNAs that mapped to exonic sequences of protein-coding genes represent degraded mRNA or resemble genuine piRNAs, the according sRNA reads were examined for piRNA characteristics. Both sense and antisense reads, which all range between 24 and 32 nt, show a strong bias for 1U (82.6% and 70.9%, respectively), while only sense sequences exhibit a marginal preference for 10A (28.2%) as compared to antisense reads (21.3%). Furthermore, piRNA reads that mapped to 115 genes exhibit a marked ping-pong signature (z-score = 22.7, Fig 3B). In addition, the length distribution of both sense and antisense cDNA-matching piRNAs reveals the presence of at least two different piRNA populations and thus the participation of different Piwi paralogs in the generation of gene-derived piRNAs (Fig 3C). Generally, piRNAs map to specific gene transcripts in a very similar fashion as compared to TE transcripts with clear signs of ping-pong-mediated amplification, which implies that mRNA is not only subject for primary processing, but can also be targeted by primary piRNAs and processed into secondary piRNAs (Fig 3D).

In order to check whether this pattern can be found in additional species, we performed the same analysis on available mouse and human sRNA and cDNA datasets. Remarkably, we observed a large amount of cDNA-matching sequences producing a clear ping-pong signature that is mainly concentrated on 185 (ping-pong-z-score = 41.2) and 424 (ping-pong-z-score = 13.4) different genes in mouse and human, respectively (Fig 3B; S2 File). Moreover, targeting of a number of gene transcripts appears to be conserved over evolutionary timescales. For instance, we noticed high piRNA coverage and ping-pong signatures on several members of the NUT (Nuclear protein in testis) gene family (NUTM2A, NUTM2B, NUTM2D, NUTM2E) for porcine as well as human piRNAs. Furthermore, ping-pong signatures were also detectable on transcripts of Histone H2A genes for all three datasets, though the read coverage is considerably lower as compared to NUT gene transcripts (S2 File). Altogether, pig and human share 15 homologous target genes (*p* = 0.0050, E(X) = 1.2188), while 7 homologs are targeted in both pig and mouse (*p* = 0.0241, E(X) = 1.0236), which are significant numbers compared to a random overlap between non-related, randomly selected genes. Hence, targeting of homologous gene transcripts across distantly related species suggests that the Piwi/piRNA system snatches mRNAs not in a random fashion but rather implies a specific biological function.

In addition, we noticed that most conserved target genes represent factors with nuclear localization that interact with DNA. Therefore we performed a GO term enrichment analysis [40] for all identified human and mouse targets with respect to the cellular component and the molecular function (S3 File, porcine data not available). Indeed, we found a significant association with the term *nucleus* for both human and mouse targets (*p* = 0.0008, *p* = 0.0055, respectively) compared to a non-significant association with the term *cytoplasm* (*p* = 1, *p* = 0.2503, respectively). Regarding the molecular function of targets we observed that human as well as mouse targets are significantly associated with the term *nucleic acid binding* (*p* = 0.0018, *p* = 0.0163). Together, these results suggest that post-transcriptional gene regulation by the Piwi/piRNA system mainly concerns nuclear factors with DNA binding activity.

### tRNA-derived sRNAs

**Fig 4:**
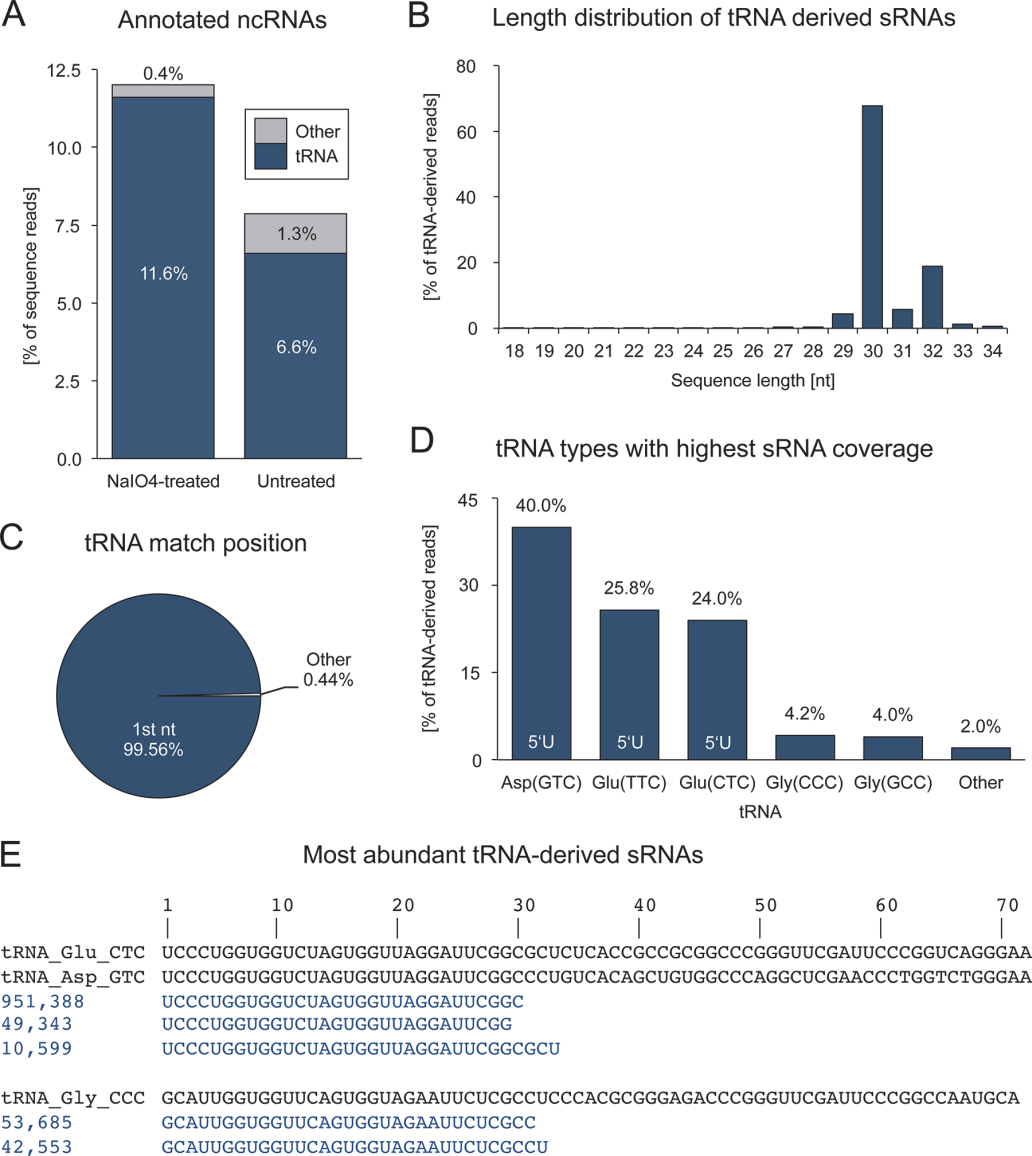
tRNA-derived small RNAs. (A) Fractions of sRNAs that were annotated as known ncRNA in a sodium periodate treated and untreated sRNA library. (B) Length distribution of tRNA-derived sRNA reads. (C) Positions on tRNAs matched by 5’ ends of sRNA reads. (D) Shares of sRNA reads mapping to distinct tRNAs. (E) Alignment of tRNA sequences and their most abundant matching sRNAs (numbers refer to read counts).

The by far largest proportion of sRNA reads that has been annotated as known ncRNA is represented by sequences that map perfectly to tRNAs and that are known as tRNA related fragments (tRFs [41], Fig 4A). Interestingly, the identified tRFs share striking similarities with piRNAs.

First, the sequence length distribution of tRNA-derived sRNAs ranges mainly from 29 to 32 nt which corresponds to the typical size of mammalian piRNAs, though we note that the length profile of the tRNA-related reads is much sharper, possibly indicating differences in biogenesis (Fig 4B).

Second, comparison with our control sRNA library without a sodium periodate treatment step reveals a less marked enrichment of tRNA-derived sequences (6.6% vs. 11.6%) while the share of other ncRNAs such as miRNAs, snRNAs, snoRNAs and rRNAs is increased (1.3% vs. 0.4%) (Fig 4A). This suggests that tRNA-derived sRNA sequences are not eliminated by sodium periodate treatment, presumably because of a modification at their 3’ end that protects them from degradation like 2’-O-methylation in case of piRNAs.

Third, tRNA-derived sequences are not randomly distributed among the various tRNA types, but rather derive mainly from the 5’ ends of five tRNA types, namely Asp-GTC, Glu-TTC, Glu-CTC, Gly-CCC, and Gly-GCC, altogether accounting for 98% of all tRNA-derived sRNA reads (Fig 4C–4E). As a consequence, about 90% of tRNA-derived reads start with a uracil. In contrast, this share reaches only 77% for the non-oxidized library with a multiple of tRNA-derived sequences that do not match the 5’ end of a tRNA (S2 Fig), which indicates the presence of random tRNA degradation products that are efficiently eliminated by periodate treatment. As opposed to 5’-end-derived reads (99.56%), only a minor share (0.01%) maps to the 3’-ends of tRNAs. In the light of the different length profiles of 5’ tRFs (18–33 nt) and 3’ tRFs (18–22 nt) [41] we suppose that the observed bias is most likely introduced by the applied cloning procedure that favors molecules larger than 24 nt.

Nonetheless, tRNA-derived sRNAs also exhibit features that clearly separate them from regular piRNAs. Interestingly, while protein-coding loci are not targeted at all, 73.5% (1,066,063 reads; 1647 non-identical sequences) of all tRNA-derived sRNA reads that map to the genome match genomic TE copies in sense (99.9%) according to RepeatMasker annotation. Not surprising, these almost exclusively represent tRNA-derived SINEs (99.1%). Finally, the share of tRNA-derived sRNA reads antisense to tRNA sequences is similarly marginal (0.002%) and a ping-pong signature is not detectable.

### Identification and characterization of piRNA clusters

Using proTRAC [35], overall 142 piRNA clusters larger than 10 kb were identified, of which 114 are unidirectional and 28 are bidirectional, altogether comprising 3.8 Mb (S4 File). These piRNA clusters are unevenly distributed across the genome, but can be found on every chromosome except for chromosomes 16 and Y (S3 Fig). The majority of total mapped sRNA reads (91%) and mapped non-identical sequences (63%) falls into the identified piRNA clusters.

**Fig 5:**
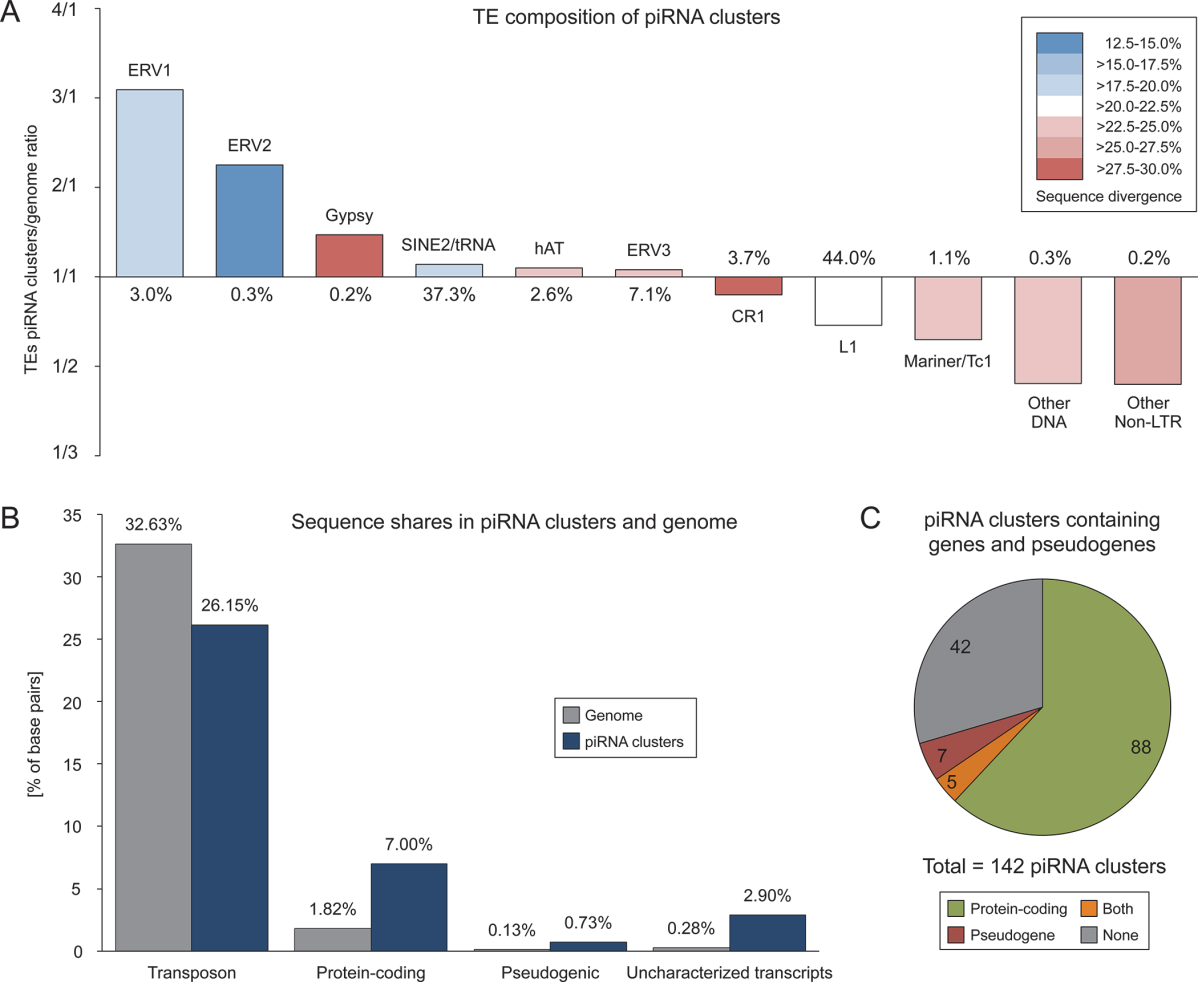
Sequence characterization of piRNA clusters. (A) TE composition of predicted piRNA clusters compared to the genomic sequence of the pig. Percentages represent the share of a TE group in the genome. A ratio above 1 indicates an enrichment of a TE group in piRNA clusters, while a ratio below 1 indicates the depletion of a TE group in piRNA clusters. Different colors express the sequence divergence of a TE group to its consensus. (B) Sequence shares of TEs, protein-coding genes, pseudogenes, and uncharacterized transcribed sequences within piRNA clusters compared to the whole genome of the pig. (C) Number of piRNA clusters containing sequences of protein-coding genes, pseudogenes or both within the same piRNA cluster.

In depth analyses of the distribution of transposon classes and families in piRNA clusters compared to the genomic situation revealed interesting differences in TE composition. ERV1 and ERV2 elements are highly overrepresented in piRNA clusters (9.1% and 0.7%) as compared to their total genomic amount (3.0% and 0.3%) (Fig 5A). At the same time, ERV1 and ERV2 elements exhibit the lowest average sequence divergence to their consensus compared to other TE classes, which implicates younger propagation events and recent activity of these elements. On the other side, CR1, L1, Mariner/Tc1, other DNA transposons and other Non-LTR elements are underrepresented in piRNA clusters, while showing a tendency for increased sequence divergence, typical for older transposon copies.

Notably, although piRNA clusters are apparently enriched for young TEs, the overall amount of transposon sequences within piRNA clusters is considerably reduced (26.2%) as compared to the whole genome (32.6%) (Fig 5B). In contrast, exonic sequences of both protein-coding genes (7.0%) and pseudogenes (0.73%) are highly enriched. Moreover, uncharacterized transcribed sequences are drastically increased in piRNA clusters (2.9%).

Overall 93 of the 142 identified piRNA clusters contain exonic sequences of protein-coding genes, while 12 contain pseudogene sequences (Fig 5C, S5 File). Only a minority of 42 piRNA clusters contains neither. We checked whether predicted piRNA clusters that span exonic sequences may simply correspond to mRNAs that are subject to primary piRNA processing. In this case we would expect piRNAs to map exons in sense orientation while no piRNAs should match to the according intronic regions. Indeed we could verify this pattern for 69 predicted piRNA clusters comprising exonic sequences that lie in sense direction of the predicted piRNA cluster and that are not producing antisense piRNAs. Since the exon-matching piRNAs also generally exhibit a high 1U rate we assume these loci to represent genes whose transcripts are processed to primary piRNAs without subsequent ping-pong amplification.

**Fig 6:**
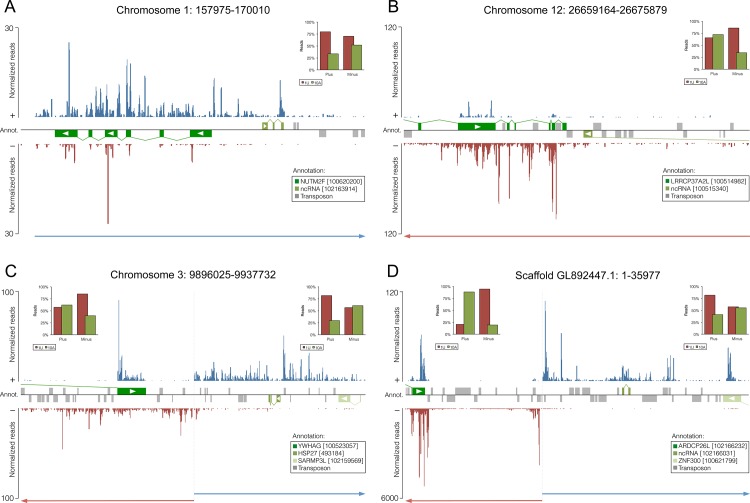
piRNA clusters containing protein-coding genes or pseudogenes. Mapping of piRNA reads on plus and minus strands of piRNA cluster sequences combined with RefSeq (NCBI) annotation of transcribed sequences and RepeatMasker annotation of TEs. NCBI GeneIDs for transcribed sequences are stated in brackets. Directions of transcription for RefSeq sequences are indicated by white arrows.

**Fig 7:**
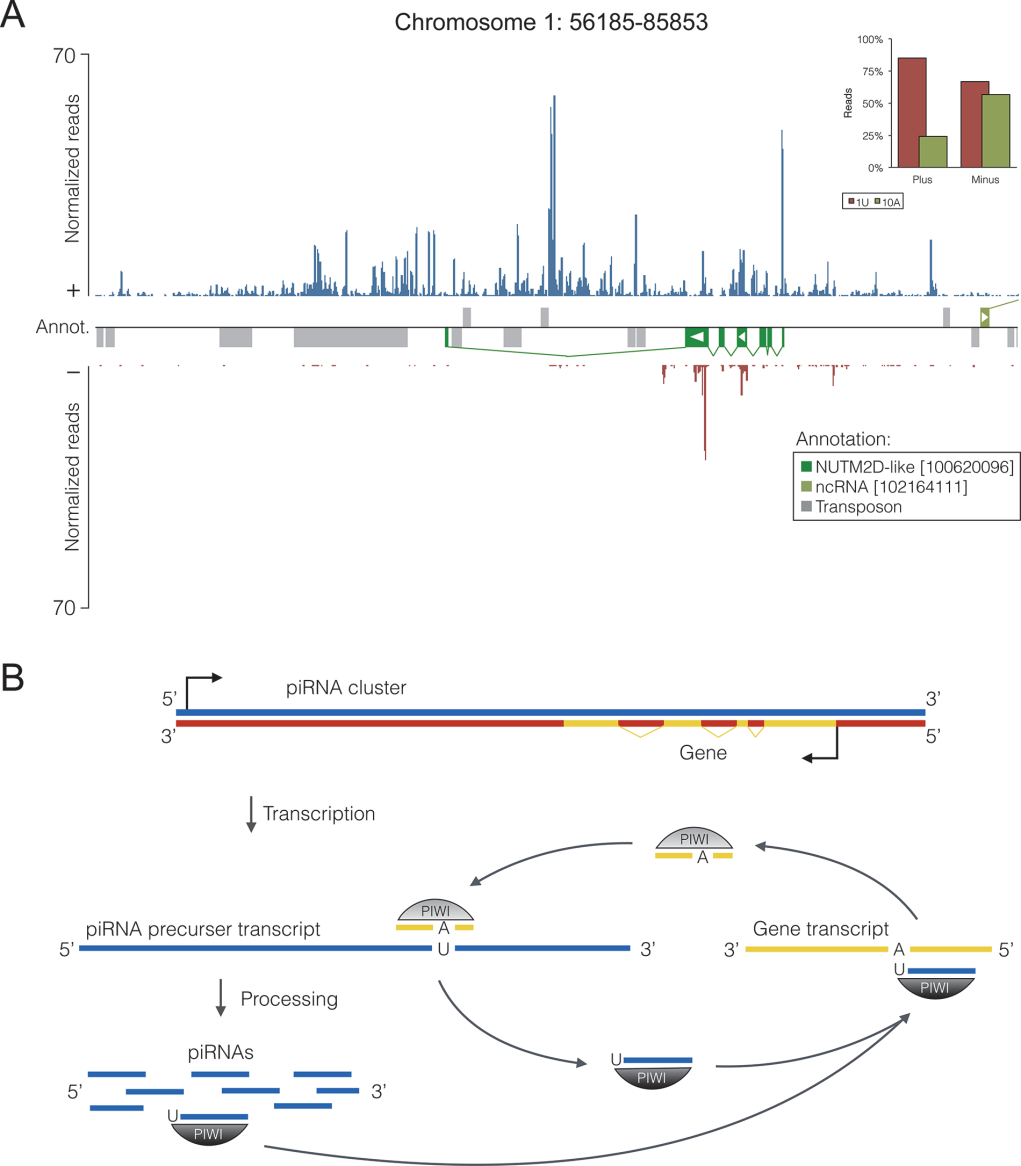
Model of post-transcriptional regulation of protein-coding genes by the Piwi/piRNA pathway. (A) piRNA cluster containing a protein-coding gene. (B) Hypothetical model of post-transcriptional gene regulation mediated by piRNA clusters, based on data of porcine piRNAs. piRNA clusters containing sequences of genes or pseudogenes in reverse orientation relative to the cluster directionality can presumably produce primary piRNAs complementary to spliced mRNA, which can direct the decay of such transcripts and produce secondary piRNAs within the ping-pong amplification loop.

Intriguingly, 62 predicted piRNA clusters comprising both, mono- and bidirectional clusters, cover protein-coding genes in opposite orientation with regards to the predicted transcription directionality of the piRNA cluster. Further, 8 out of 12 pseudogenes within piRNA clusters are oriented in antisense direction relative to the main strand of the piRNA cluster. While piRNA reads mapping to the main strand, which corresponds to the putative primary piRNA cluster transcript, are distributed across the entire piRNA cluster sequence, piRNAs matching the opposite strand are largely restricted to the exonic regions of the corresponding overlapping gene (Fig 6A–6D and 7A). Notably, the latter generally exhibit a reduced 1U rate but an increased 10A rate as compared to main strand reads. These data strongly suggest that primary antisense piRNAs produced from these loci are targeting spliced transcripts of genes that are transcribed from the opposite strand, and that this targeting is followed by secondary piRNA biogenesis (Fig 7B).

Overall 24% of the piRNA reads that match porcine cDNA sequences originate from predicted piRNA clusters. Interestingly, cDNA-matching piRNA reads that lie outside of piRNA clusters are strongly biased towards sense sequences (88%) indicating mainly primary processing of the according transcripts. In contrast, cDNA-derived reads that can be assigned to piRNA clusters exhibit a nearly balanced ratio of sense versus antisense reads (55% and 45%, respectively). This points to a central role of piRNA clusters in the processing of specific protein-coding gene transcripts within the ping-pong cycle of the Piwi/piRNA pathway.

## Discussion

Studies on model organisms like *Drosophila* and mouse have been highly informative relating to the functions and the molecular mechanisms of the Piwi/piRNA pathway. However, these organisms do not reflect the equipment of Piwi paralogs in most mammals, including human [26]. In this respect, the pig with its full set of four mammalian Piwi paralogs is more comparable to humans. Furthermore, the availability of a high quality porcine genome assembly combined with a thorough annotation of porcine TEs, along with powerful molecular biological tools [42,43] render the pig a suitable model for Piwi/piRNA research. Our extensive characterization of the porcine piRNA transcriptome represents the initial step on the way to understand piRNA function in the pig and to obtain a broader knowledge of the Piwi/piRNA pathway in mammals.

Considering their main features, porcine piRNAs closely reflect previously described characteristics of mammalian piRNAs. The length distribution of pig piRNAs ranges mainly from 24–32 nt, though the majority of 20–25 nt sized sRNAs was also found to exhibit typical piRNA characteristics and could be mapped to predicted piRNA clusters, thus most likely representing genuine piRNAs rather than non-oxidized siRNAs. Further, porcine piRNAs expressed in the adult testis show a strong bias for 1U and only a marginal bias for 10A, suggesting that the bulk originates from primary processing, while only a small fraction results from ping-pong amplification. However, while previous studies on porcine piRNAs did not report any ping-pong signatures [28,29], they are apparent in our data, clearly demonstrating that ping-pong-mediated silencing is active also in the adult germline. Moreover, though a recent study on porcine piRNAs reported the absence of ping-pong signatures [29], we could on the contrary validate our findings (ping-pong-z-score = 8.7) using the data produced by Kowalczykiewicz and colleagues (NCBI Gene Expression Omnibus (GEO); accession number GSE57414). Interestingly, we could also show a ping-pong signature in the corresponding sRNA dataset obtained from pig ovaries (ping-pong-z-score = 28.5), though on a very low level (S6 File). In line with this, piRNA expression and ping-pong-signatures in the female germline were also very recently described in human, macaque and bovine piRNA populations by Roovers and coworkers [44].

The analyzed piRNAs in our study, isolated from whole testes and thus representing a mixture of piRNAs from all germ cell stages (pachytene and pre-pachytene), are clearly depleted of TE-related sequences compared to the total genomic amount of TEs in pig. This is in line with findings from the mouse model in that only meiotically (pre-pachytene) expressed piRNAs are enriched for TE-related sequences and participate in the ping-pong cycle to repress TEs that become active during global de- and re-methylation in spermatogenesis [9,13]. This gives rise to the question whether piRNAs, especially in pachytene stages may be involved in functions beyond TE silencing.

### tRNA-derived sRNAs with piRNA characteristics

piRNAs exhibit a methylation of the 2’-hydroxyl group at their 3’ end and are therefore protected from sodium periodate-mediated ß-elimination [45,46]. RNA molecules lacking this modification are removed during library preparation [47]. Comparing oxidized and non-oxidized libraries, we noted an enrichment of tRNA-related sRNAs after periodate treatment, while sRNAs related to other ncRNA types almost completely disappeared. This suggests that they may also carry a 3’ methylation similar to piRNAs that prevents their decay. Indeed, methylation of tRNA nucleotides is a common phenomenon and 2’-O-methylation of nucleotides 30 to 32 is described for tRNAs of many mammalian species [48], although data on porcine tRNAs is lacking.

Another interesting characteristic is that nearly all tRNA-derived sRNAs originate from the 5’ ends of only five different tRNA types, with the majority of them starting with a uracil. tRFs [41], such as the 5’ tRNA halves that we describe here, along other types of short fragments of tRNAs like 5’ tRFs, 3’ tRFs and 3’ tRNA halves have been previously found in many different species [49–52,38]. Presumably, 5’ tRNA halves are produced by a conserved stress response mechanism in eukaryotes [53] and play a role in translational regulation [54], as well as impact the siRNA pathway by inhibiting Dicer activity [55]. Some 5’ tRFs have been shown to be produced by Dicer, bound by Argonaute proteins and further to carry blocked 2’ hydroxyl termini [56]. With regard to their biological role, 5’ tRFs have been implicated in gene regulation [57], e.g. by inhibition of protein translation, which does not require complementary base pairing [58]. Also, tRFs have been reported very recently to be present in male and female gonads of the pig [29], although the composition of tRNA types differed notably from our results.

Recently, the Piwil1 homolog Marwi of the common marmoset has been found to bind considerable amounts of tRNA-derived sRNAs, which exhibit very similar characteristics as described here [59]. Furthermore, various tRFs associate with the human Piwil2 homolog Hiwi2 [60] and the *Tetrahymena* Piwi Twil2 [61]. In addition, short tRNA sequences have been previously described as piRNAs in several organisms such as rat, human [4], mouse [62] and hamster [63].

We speculate that generally all tRNAs should be subject to a processing mechanism that yields 5’ tRFs but that Piwi proteins are loaded only with 1U fragments that a priori carry a 3’ methylation as do the corresponding tRNAs. Therefore, we hypothesize that the described tRNA-derived sRNAs literally represent piRNAs in that they interact with Piwi proteins. However, since we did neither detect a ping-pong signature nor identified putative complementary target transcripts, their biological role, if any, may be limited to functions that are not related to the Piwi pathway.

### Repression of transposable elements

Silencing of transposons is regarded as the major task of piRNAs in the animal germline [19] and a considerable amount of porcine piRNA sequences indeed maps to TE sequences. Consistent with the fact that the share of TE sequences in the porcine genome is lower than reported for other mammalian genomes [27], the proportion of TE-derived piRNAs is likewise reduced with respect to other species.

The elevated shares of piRNAs mapping to tRNA-derived SINEs and especially to ERVs compared to the genomic amount of these elements might reflect a recent activity of these transposon classes in the porcine genome. Indeed, ERV1 elements have been found to show hints of recent activity on the pig lineage and an increased insertion rate at pig specific evolutionary breakpoint regions [27], while tRNA^Glu^-derived SINEs, a cetartiodactyl specific TE superfamily [64], have been found to be overrepresented in cetartiodactyl evolutionary breakpoint regions [65]. What further supports the presumption of a recent activity is the fact that ERV1, ERV3 and tRNA-derived SINEs show the least sequence divergence to their consensus compared to other TE classes, pointing to a younger age and more recent activity. These TEs, foremost ERV1, are also enriched in the predicted piRNA clusters identified here. This suggests not only that the Piwi/piRNA system is highly adaptable, but it also might indicate that piRNA clusters can act more dynamically and/or selectively than commonly thought.

Hypothetically, new piRNA clusters might emerge at sites with a high rate of recent integrations of active TEs. On the other hand, since piRNA clusters represent transcriptionally highly active regions in the genome, non-inert TEs might more likely integrate into such regions than into sites that have a more closed chromatin structure. Contrasting this intuitive assumption, piRNA clusters are not enriched for TEs, but on the contrary are poorer of TE sequences compared to the remaining genome. Apparently there must be either an efficient TE insertion avoidance mechanism or alternatively natural selection against the accumulation of TEs into piRNA clusters which could explain the general bias towards non-TE sequences.

### Regulation of protein-coding genes

The first identification of piRNAs derived from protein-coding genes dates back to the initial description of piRNAs [4–6], but a regulatory role was not considered even in following studies [14] due to a lack of antisense piRNAs. A later report showed that the 3’ untranslated regions (3’ UTRs) of a set of mRNAs in murine testes are processed into primary piRNAs, while no secondary piRNAs or signs of ping-pong processing could be observed [24]. Indeed, we confirm that the mapping density (reads per kb) of porcine piRNAs on cDNA is highest on 3’ UTRs, which however can be partly explained by the fact, that 3’ UTRs are enriched for TE sequences compared to 5’ UTR and coding sequence, though the share of TE-related piRNA reads mapping to 5’- and 3’ UTRs does not differ substantially (S4 Fig).

In this study we found that both sense and antisense piRNAs map to exonic sequences of protein-coding genes, showing marked ping-pong signatures resulting from sense and antisense reads derived from mRNA sequences of a large number of genes. Moreover, the length distribution of exon-derived piRNAs indicates the participation of different Piwi paralogs in their generation. Together, this suggests that gene transcripts are processed into piRNAs within the ping-pong cycle.

A central role for this process, as known for TEs, seems to be occupied by piRNA clusters. piRNAs mapping to both strands at exonic regions of piRNA clusters that span genes in reverse direction, as well as their opposing 1U and 10A rates suggest that piRNAs antisense to the corresponding gene are produced in primary biogenesis from large cluster transcripts. These primary piRNAs can in turn guide the piRNA-induced silencing complex (piRISC) machinery to target mRNAs that enter the ping-pong cycle to generate secondary sense piRNAs (Fig 7B). In support of this model, the majority of antisense gene-related reads derives from piRNA clusters, although only a quarter of all gene-derived reads can be assigned to piRNA clusters. Overall, these observations reveal a mechanism by which antisense piRNAs are produced to direct mRNA processing and exert Piwi-mediated post-transcriptional regulation on protein-coding genes.

Finally, the fact that specific genes are targeted not only in pig but also in human and mouse suggests a conserved biological function during eutherian divergence. In support of this, GO term enrichment analysis revealed that targeted genes mainly represent factors with nuclear localization and DNA binding activity, suggesting their involvement in transcriptional regulation and chromatin modification. These results strengthen findings from a previous study on porcine piRNAs that revealed similar patterns regarding possible piRNA target genes but lacks a quest for ping-pong signatures [28].

Whether the processing of gene transcripts by the Piwi/piRNA pathway, foremost within the ping-pong cycle, has a significant effect on transcription levels yet has to be investigated. However, it is likewise conceivable that target genes are not extensively silenced, but rather experience a fine-tuning of their expression. The specific role of targeted transcripts in spermatogenesis is yet unresolved. Though many of the highly targeted transcripts in human such as DNM1P46, GOLGA2P11, NPAP1P6 or FBXO25 are exclusively or mainly expressed in testis according to Expression Atlas data [66], evidence for an involvement in spermatogenesis is generally lacking. One exception is the NPAP1 gene (alias c15orf2) which has been linked to spermatogenesis and male infertility in human [67].

Our findings line up into a range of results from previous studies on mammalian piRNAs and reinforce the idea that piRNAs are involved in post-transcriptional gene regulation. Recently, it has been demonstrated that pachytene piRNAs direct mRNA elimination during late spermatogenesis in mouse [25]. Importantly, a very recent study [68] led to observations similar to ours regarding ping-pong-mediated mRNA processing in mouse testis. It further showed that the proper turnover of certain key piRNA targets seems to be essential for sperm formation, strengthening the concept of an important role for the Piwi pathway in the regulation of protein-coding genes.

Moreover, analyses of testis expressed piRNAs from the common marmoset also showed that pseudogenes are located in piRNA clusters and tend to be in reverse orientation relative to piRNA cluster directionalities [59]. However, these pseudogenic regions were only covered by piRNAs on one strand, whereas one would expect signs of a ping-pong signature if these piRNAs would participate in Piwi-mediated silencing of the corresponding genes. Going back to the initial description of testis expressed piRNAs in mouse, protein-coding genes have been found to overlap with piRNA cluster sequences, though possible gene regulatory functions were ruled out because of a lack of gene-derived antisense piRNAs [6]. Nevertheless, the existence of piRNA clusters containing gene or pseudogene sequences is not pig specific, but likely a widespread phenomenon.

Interestingly, antisense transcripts for NUTM2A (lncRNA), NUTM2B (lncRNA) and NUTM2D (ncRNA) and other target genes are predicted for human according to the HAVANA genome annotation. In addition, though only very few porcine lncRNAs are annotated, sRNA reads derived from such sequences show clear piRNA characteristics, such as a marked ping-pong signature and 1U and 10A bias (S5 Fig). Concordantly, putative piRNAs have been recently found to map to lncRNA sequences in humans [37]. This suggests that (long) non-coding RNAs are processed into primary piRNAs or alternatively represent primary piRNA cluster transcripts, which appears to be rather a matter of definition.

In summary, the enrichment of protein-coding gene sequences together with the evidence for their ping-pong-mediated post-transcriptional processing, and the presence of rather young transposon classes accompanied by an overall reduced amount of transposons in piRNA clusters challenge the model of passive transposon traps. Extending this traditional view, we consider it possible that piRNA clusters might specifically arise at genomic loci whose transcripts (protein-coding or not) require control by the Piwi/piRNA system, yielding a beneficial, positively selectable mechanism for the host organism. Clearly, this hypothesis has to be further addressed in the future.

## Supporting Information

S1 Fig Directionalities of TE sequences in piRNA clusters and TE-derived piRNA reads. Correlation between insertion bias of TE copies and strand bias of TE-related piRNAs for the TE classes with highest read coverage, tRNA-derived SINEs, L1, and ERV1.
(TIF)

S2 Fig Periodate treatment of tRNA-derived small RNAs. Comparison of tRNA-derived sRNAs from NaIO4-treated and untreated libraries. (A) and (B) Shares of sRNA reads mapping to distinct tRNAs. tRNAs that possess a 5’ uracil are marked with 1U. (C) and (D) Positions on tRNAs matched by 5’ ends of sRNA reads and 1U rates of tRNA-derived reads. (E) and (F) Length Distribution of tRNA-derived sRNA reads.
(TIF)

S3 Fig Distribution of piRNA clusters and piRNAs in the porcine genome. (A) Genomic piRNA cluster distribution. Red dashes mark the position of a cluster on a chromosome (gray thick bars), counting from top to bottom. Thin blue bars represent the total number of clusters per chromosome. (B) Shares of reads and non-identical sequences of piRNAs mapping to piRNA clusters.
(TIF)

S4 Fig Small RNA reads derived from 5’UTR, CDS and 3’UTR. (A) Relationship between piRNA mapping bias and TE enrichment of 3’UTRs. (B) 5’ overlaps of piRNAs derived from 5’UTRs, CDS and 3’UTRs. (C) Length distributions of piRNAs derived from 5’UTRs, CDS and 3’UTRs.
(TIF)

S5 Fig Characterization of sRNA reads derived from annotated porcine lncRNAs. (A) Length distribution of sense and antisense sRNA reads. (B) 5’ overlaps of sRNA reads. (C) 1U and 10 A rates of sense and antisense sRNA reads.
(TIF)

S1 File 5’ overlaps and z-scores for all mapped, TE-related and cDNA-related reads. (XLSX)

S2 File Target genes and ping-pong signatures of gene-derived small RNAs in pig, mouse, and human. (XLSX)

S3 File GO term enrichment results for ping-pong target genes of mouse and human. (XLSX)

S4 File Annotation of pig piRNA clusters according to proTRAC. (XLSX)

S5 File Annotation of RefSeq gene and pseudogene sequences within pig piRNA clusters. (XLSX)

S6 File Analyses of porcine small RNA data from Kowalczykiewicz et al. 2014. (XLSX)
