# Supplementary figures and images for: piRNAs from Pig Testis Provide Evidence for a Conserved Role of the Piwi Pathway in Post-Transcriptional Gene Regulation in Mammals
Source: PLoS One. 2015 May 7;10(5):e0124860. doi: 10.1371/journal.pone.0124860 (PMC4423968; doi:10.1371/journal.pone.0124860)

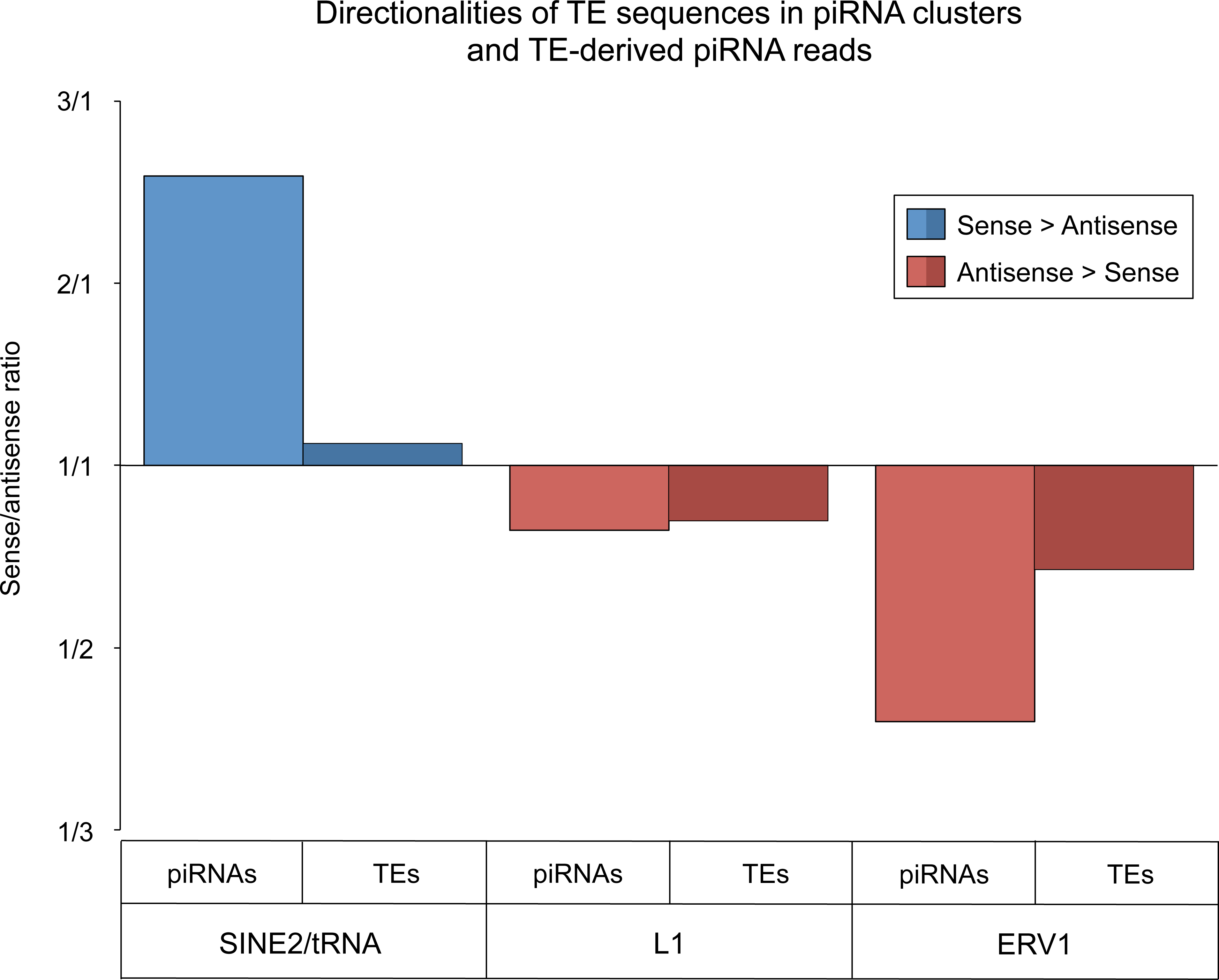

Supplement: S1 Fig — Correlation between insertion bias of TE copies and strand bias of TE-related piRNAs for the TE classes with highest read coverage, tRNA-derived SINEs, L1, and ERV1. (TIF) [file pone.0124860.s001.tif]

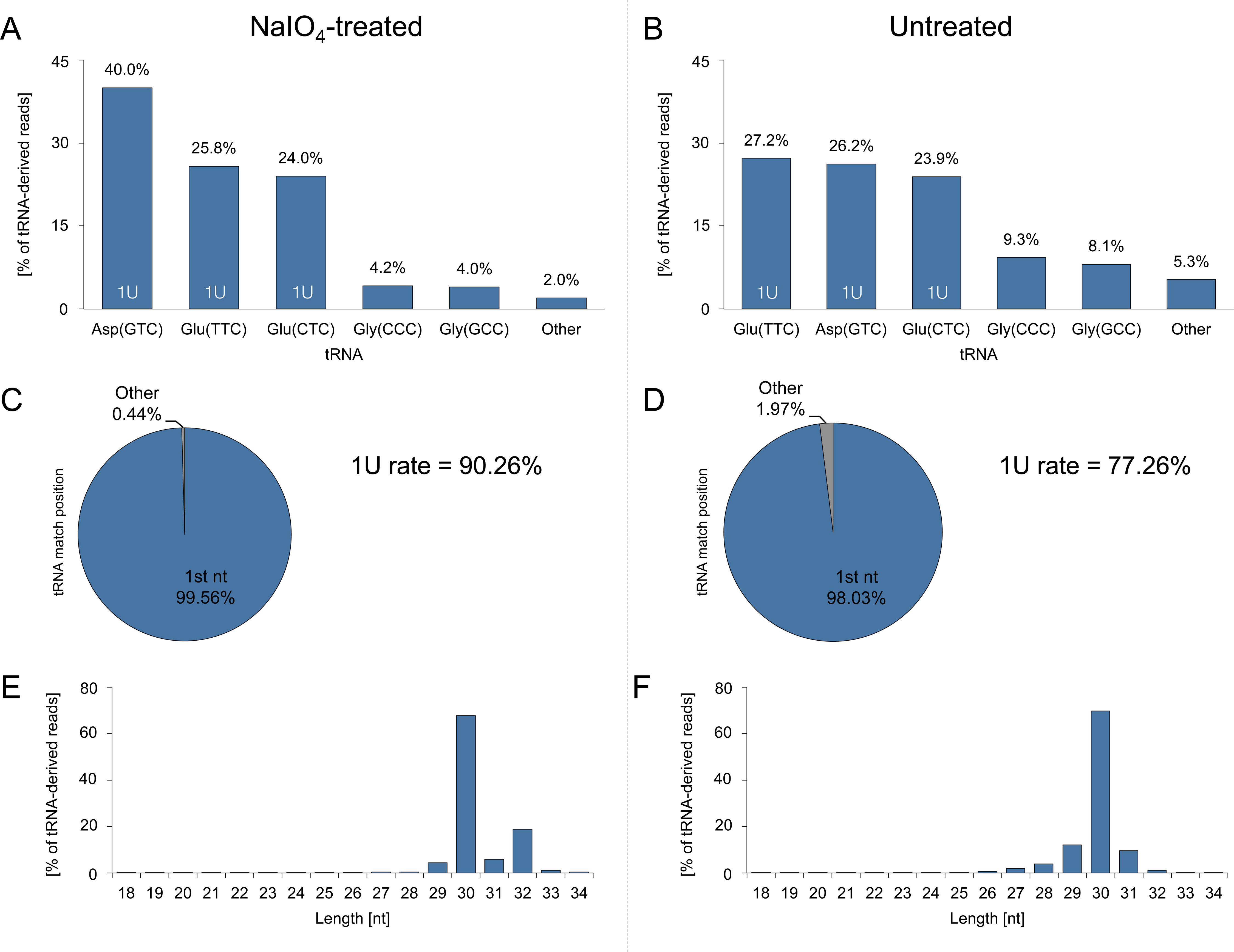

Supplement: S2 Fig — Comparison of tRNA-derived sRNAs from NaIO4-treated and untreated libraries. (A) and (B) Shares of sRNA reads mapping to distinct tRNAs. tRNAs that possess a 5’ uracil are marked with 1U. (C) and (D) Positions on tRNAs matched by 5’ ends of sRNA reads and 1U rates of tRNA-derived reads. (E) and (F) Length Distribution of tRNA-derived sRNA reads. (TIF) [file pone.0124860.s002.tif]

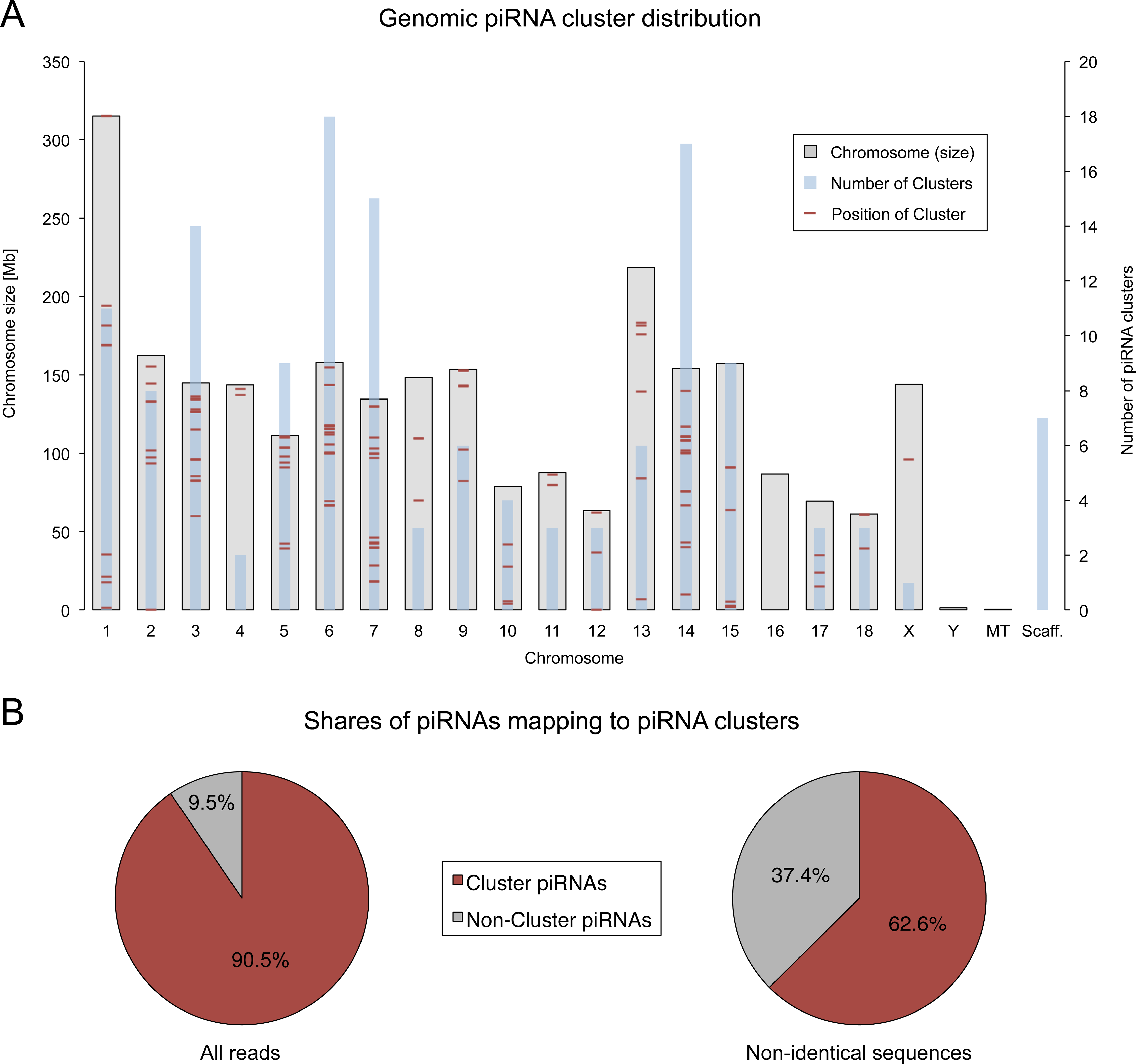

Supplement: S3 Fig — (A) Genomic piRNA cluster distribution. Red dashes mark the position of a cluster on a chromosome (gray thick bars), counting from top to bottom. Thin blue bars represent the total number of clusters per chromosome. (B) Shares of reads and non-identical sequences of piRNAs mapping to piRNA clusters. (TIF) [file pone.0124860.s003.tif]

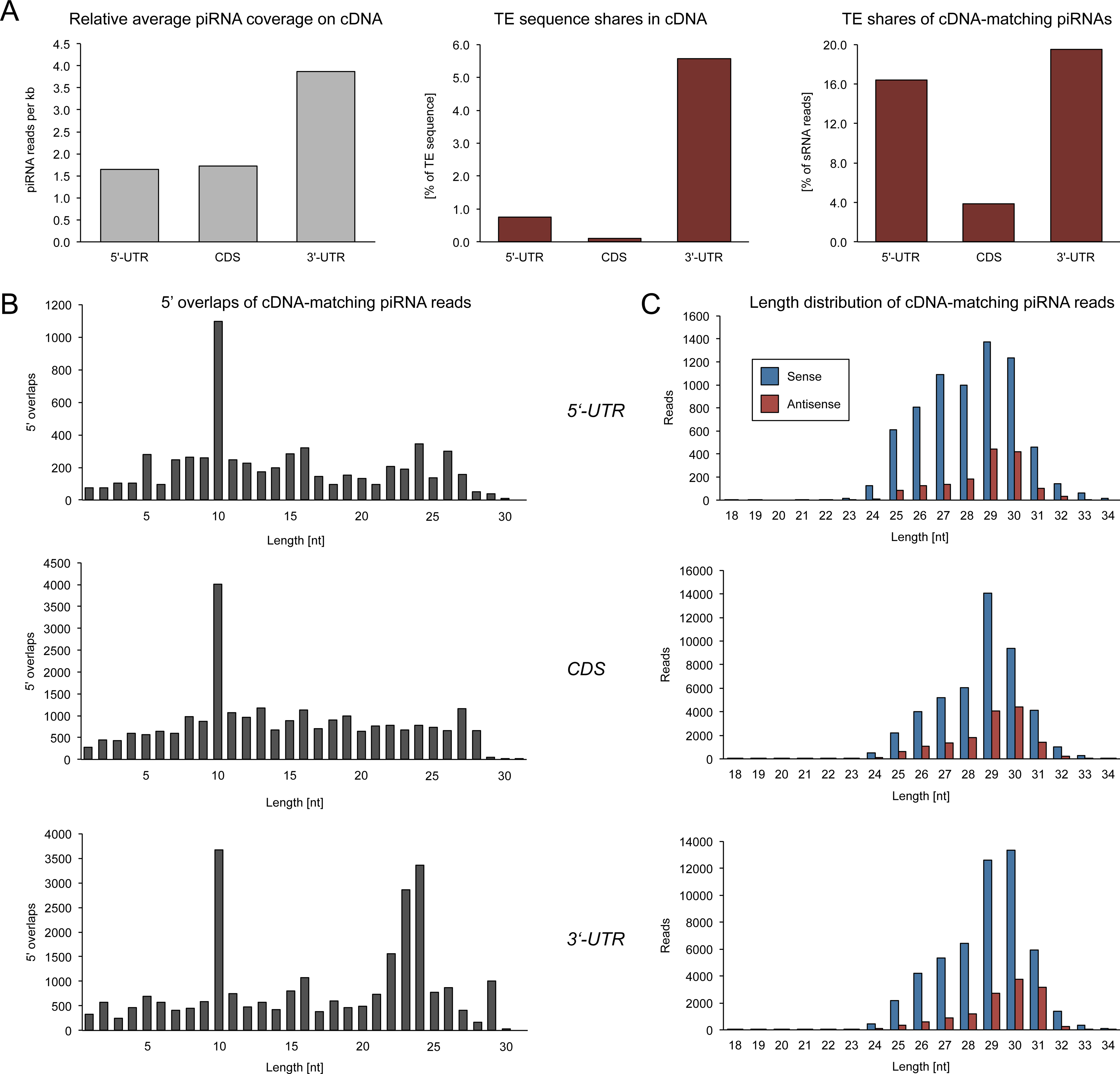

Supplement: S4 Fig — (A) Relationship between piRNA mapping bias and TE enrichment of 3’UTRs. (B) 5’ overlaps of piRNAs derived from 5’UTRs, CDS and 3’UTRs. (C) Length distributions of piRNAs derived from 5’UTRs, CDS and 3’UTRs. (TIF) [file pone.0124860.s004.tif]

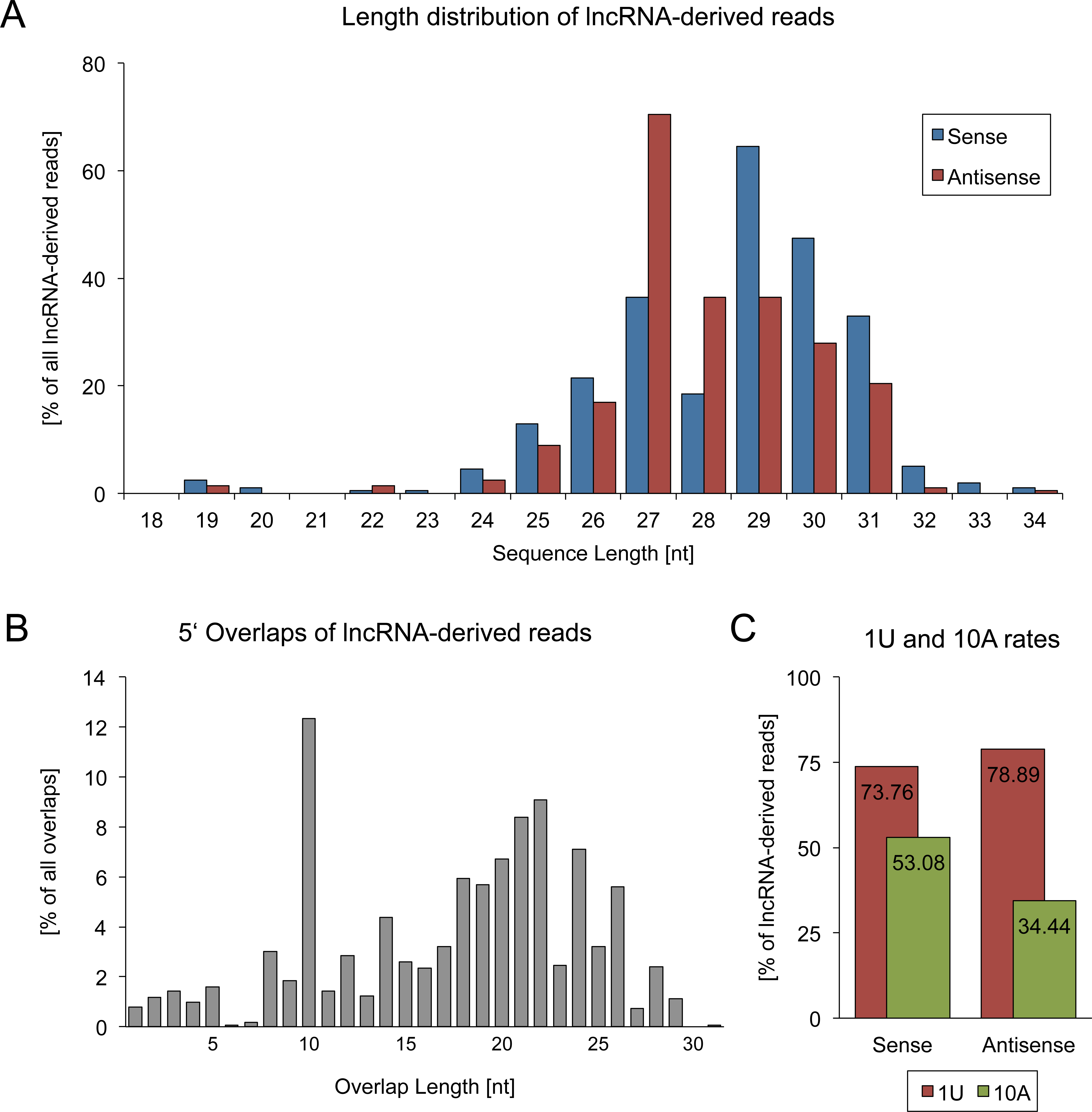

Supplement: S5 Fig — (A) Length distribution of sense and antisense sRNA reads. (B) 5’ overlaps of sRNA reads. (C) 1U and 10 A rates of sense and antisense sRNA reads. (TIF) [file pone.0124860.s005.tif]
